# MicroRNA-203 Is a Prognostic Indicator in Bladder Cancer and Enhances Chemosensitivity to Cisplatin via Apoptosis by Targeting Bcl-w and Survivin

**DOI:** 10.1371/journal.pone.0143441

**Published:** 2015-11-23

**Authors:** Xin Zhang, Yanli Zhang, Xinfeng Liu, Aiju Fang, Peilong Li, Zewu Li, Tong Liu, Yongmei Yang, Lutao Du, Chuanxin Wang

**Affiliations:** 1 Department of Clinical Laboratory, Qilu Hospital, Shandong University, Jinan, China; 2 Department of Clinical Laboratory, Traffic Hospital of Shandong Province, Jinan, China; 3 Department of Pathology, Traffic Hospital of Shandong Province, Jinan, China; IPMC, CNRS UMR 7275 UNS, FRANCE

## Abstract

Resistance to cisplatin-based chemotherapy is a major cause of treatment failure in advanced bladder cancer (BC) patients. There is increasing evidence that microRNAs are involved in the development and progression of BC. However, little is known about the function of microRNAs in predicting the effect of adjuvant chemotherapy on BC survival and regulating response to cisplatin. To address this issue, we employed RT-qPCR to evaluate the clinical significance of miR-203 expression in 108 tissues of BC patients receiving cisplatin-based adjuvant chemotherapy, and performed in vitro studies to explore chemotherapeutic sensitivity to cisplatin in miR-203 overexpressing BC cells. We found miR-203 levels were significantly lower in BC progression group than non-progression group (*P*<0.001). ROC curve analysis illustrated miR-203 could significantly distinguish progressed patients from those without progression (*P*<0.001), yielding an area under the ROC curve of 0.839 (95% CI, 0.756–0.903). Moreover, low miR-203 expression correlated with shortened progression free survival (PFS) and overall survival (OS) of BC patients, and was an independent prognostic factor. Overexpression of miR-203 in 5637 and T24 BC cells could decrease cell viability, enhance cisplatin cytotoxicity, and promote apoptosis. Western blotting and luciferase reporter assay showed Bcl-w and Survivin were direct downstream targets of miR-203. There was also a significant inverse association between miR-203 and Bcl-w or Survivin expression in BC tissues (r = -0.781, -0.740, both *P*<0.001). In conclusion, decreased miR-203 predicts progression and poor prognosis for BC patients treated with cisplatin-based chemotherapy while miR-203 overexpression can enhance cisplatin sensitization by promoting apoptosis via directly targeting Bcl-w and Survivin.

## Introduction

Worldwide, bladder cancer (BC) is the second most common malignancy of the urinary system, with about 386,300 new cases and 150,200 deaths annually[1]. Because most locally advanced BC patients experience relapse after radical cystectomy[2], adjuvant chemotherapy is usually preformed in an effort to delay recurrence and prolong survival. Presently, cisplatin is one of the most important chemotherapy drugs in BC combination regimen, such as MVAC (methotrexate, vinblastine, doxorubicin, and cisplatin) and GC (gemcitabine and cisplatin). However, only 50% of muscle invasive BC patients have responded to cisplatin-based chemotherapy[3]. Even worse, some patients only suffer toxicity without achieving chemotherapeutic benefit. Therefore, there is an urgent need to predict the effect of adjuvant chemotherapy on BC survival and understand the mechanisms that prevent response to chemotherapy.

Recent studies have found resistance to cisplatin treatment could be mediated by microRNAs (miRNAs) [4, 5]. miRNAs are a class of non-coding regulatory RNAs composed of approximately 22 nucleotides which function primarily to downregulate target mRNAs by specifically binding to their 3’-untranslated region (3’-UTR) and subsequently promoting degradation and/or inhibiting translation[6, 7]. Multiple publications have documented miRNAs can act as tumor suppressors or oncogenes involved in tumor formation, maintenance, and metastasis, and are potential biomarkers for cancer diagnosis, therapeutic outcome and prognosis [8–10]. Among these, miR-203 usually acts as a tumor suppressor, and is down-regulated in multiple types of human malignancies including BC [11]. Recently, miR-203 expression has been linked to the development of resistance against chemotherapy in many cancers. For example, miR-203 levels were decreased in acquired chemotherapy drug–resistant breast cancer cells[12]. Overexpression of miR-203 enhanced the anticancer effect of paclitaxel in colon cancer cells through inhibiting cell proliferation, promoting cell apoptosis and death[13]. In contrast, Zhou et al [14] found exogenous expression of miR-203 induced resistance to oxaliplatin in colorectal cancer cells. Until now, there is little known about the potential role of miR-203 in cisplatin-based BC chemotherapy and further research is needed.

In this study, miR-203 was examined in clinically resected tissues of BC treated with radical cystectomy and cisplatin-based adjuvant chemotherapy, and the association between miR-203 and prognosis was analyzed. Furthermore, the mechanisms underlying the role of miR-203 in chemoresistance of BC were investigated. We found low expression of miR-203 was correlated with progression and poor survival of BC patients receiving cisplatin-based adjuvant chemotherapy. Furthermore, restoration of miR-203 expression could enhance sensitivity of cisplatin in BC cells through promoting cell apoptosis by targeting Bcl-w (also known as BCL2L2) and Survivin (also known as BIRC5), which indicated miR-203 might have some potential value in prognosis prediction and therapeutic application.

## Materials and Methods

### Patients and tissue samples

The study was approved by the Ethics Committee of Qilu Hospital of Shandong University, and written informed consent from each patient was also obtained. The initial study included a total of 149 BC patients treated with radical cystectomy and cisplatin-based adjuvant chemotherapy in Qilu Hospital, Shandong University between April 2007 and March 2010. All patients had histologically confirmed localized transitional cell carcinoma, staged as pT3a-4a/N+ and M0 according to 2010 AJCC/TNM classification. The standard chemotherapeutic regimen (MVAC or GC) was given between 3 to 10 weeks after complete resection, with a maximum of 6 cycles unless progression or unacceptable toxicity appeared. Of these, 25 received radiotherapy, BCG treatment or neoadjuvant chemotherapy, and 16 were excluded due to incomplete resection. The remanding 108 patients were followed up with cystoscopy quarterly for the first 2 years, then semiannually to 5 years, and annually thereafter until January 2015. Progression was defined as locoregional recurrence, distant metastasis, or death due to BC. Progression free survival (PFS) was calculated from the time of surgery to the date of first progression, and Overall survival (OS) was calculated from the time of surgery to the date of death. Patients without above event were censored at study completion. All tissues were snap-frozen and confirmed by pathological analysis, and then stored at -80°C until RNA extraction.

### Cell lines and culture

The human BC cell lines (5637 and T24) and HEK 293T cell line were purchased from American Type Culture Collection (Manassas, VA, USA), and maintained in RPMI 1640 (Thermo Fisher Scientific) supplemented with 10% fetal bovine serum (Sigma-Aldrich, St. Louis, MO, USA) and cultured at 37°C with a humidified environment of 5% CO_2_ in air.

### Transfection

Lipofectamine 2000 (Invitrogen, Carlsbad, CA, USA) was used for miR-203 mimics / miRNA mimics negative control transfections according to manufacturer’s instructions. miR-203 mimics (Dharmacon, Lafayette, CO, USA) were double-stranded RNA oligonucleotides designed to mimic endogenous, mature miR-203. miRNA mimics negative control (Dharmacon) was based on cel-miR-67, mature sequence: UCACAACCUCCUAGAAAGAGUAGA, and had identical design and modifications as miR-203 mimics.

### RNA extraction and RT-qPCR

Total RNA was extracted from tissues using standard TRIzol method (Invitrogen, Carlsbad, CA) following the manufacturer’s protocol, and measured by NanoDrop spectrophotometer (Thermo Fisher Scientific, Waltham, MA, USA). RT-qPCR was performed in ABI PRISM 7500 Sequence Detection System (Applied Biosystems). Detection of miR-203 was carried out as described previously[15]. For Bcl-w and Survivin mRNAs quantification, RNA was reverse-transcribed using High Capacity cDNA Reverse Transcription Kit (Applied Biosystems, Foster City, CA, USA), then the 10-fold diluted cDNA was amplified with Power SYBR Green PCR Master Mix (Applied Biosystems). The relative expression level of miR-203 and Bcl-w and Survivin mRNAs were calculated using 2^−ΔΔCT^ method through normalized to reference genes U6 snRNA and GAPDH mRNA, respectively.

### Cell viability assay

Cell viability was quantified using the Cell Counting Kit-8 (CCK-8; Diojindo Laboratories, Kumamoto, Japan) according to the manufacturer’s instructions. Cells (5×10^3^ cells/ well) were seeded in 96-well plates in triplicate 24h after transfection, and incubated with 0, 5, 10, 15, 20, 25 and 30μM cisplatin in 100 μl culture medium for another 24h. Then, WST-8 substrate was added at 37°C for 2h, and absorbance was read by spectrophotometer at 450nm.

### Flow cytometry for apoptosis assay

Flow cytometry analysis of apoptosis was analyzed using an Annexin V-FITC/PI staining kit (BestBio, Shanghai, China) according to the manufacturer’s instructions. Twenty-four hours after transfection, cells (1×10^4^ cells/ well) were stimulated with 5μM cisplatin for 48h and then collected. After washing 3 times with with cold PBS, the cells were resuspended in binding buffer followed by staining with Annexin V-FITC for 15min and PI for 5min in darkness. 1× 10^4^ cells were assessed on a FACS Calibur flow cytometer (BD, Bedford, MA, USA). All experiments were done in triplicate.

### TUNEL assay

Cells (1×10^4^ cells/ well) were seeded in 96-well plates in triplicate after transfection, and incubated with 5μM cisplatin in 100μl culture medium for 24h. Cells were fixed in 4% paraformaldehyde for 30 min, then neutralized with 2mg/ml Glycine for 5min, permeabilized in 0.1% Triton X-100 for 10min, and finally labeled with fluorescein-12-dUTP using terminal deoxynucleotidyl transferase. The localized green fluorescence of apoptotic cells (fluorescein-12-dUTP) was detected by fluorescence microscopy.

### Western blotting analysis

Forty‐eight hours after transfection, the cells were lysed in ice-cold radio immunoprecipitation assay (RIPA) buffer for 30min, and the protein concentration was quantified using Bio-Rad protein assay reagent (Bio-Rad, CA, USA). Thirty μg protein samples were loaded and separated on 10% SDS–polyacrylamide gels, and transferred to polyvinylidene fluoride membranes (Millipore, Bedford, MA, USA). The membranes were first blocked with 5% non-fat skim milk for 2h, then incubated with anti-Bcl-w (1:1000; Cell Signaling Technology, Beverly, MA, USA), anti-Survivin (1:1000; Abcam, Cambridge, Massachusetts, USA) or anti-β-actin rabbit monoclonal antibody (1:1000; Cell Signaling Technology) overnight at 4°C, followed by incubation with HRP-labeled secondary antibody (1:5000; Cell Signaling Technology) for 1h at room temperature. Finally, the bands were visualized using chemiluminescence detection kit (Amersham Pharmacia Biotech, Piscataway, NJ, USA) on FluorChem E Chemiluminescent Western Blot Imaging System (Cell Biosciences, Santa Clara, CA, USA).

### Luciferase reporter assay

The pmiR-REPORT^TM^ vectors (RiboBio, Guangzhou, China) were constructed with wild type (WT) or mutant (MUT) 3'-UTR of Bcl-w and Survivin, and all sequences were inserted between the hRluc and the hLuc gene. HEK293T cells (1×10^4^ cells/ well) were seeded in 96-well plates and co-transfected with miR-203/ negative control miRNA and WT-Survivin 3'-UTR vector/MUT- Survivin 3'-UTR vector or WT-Bcl-w 3'-UTR vector/MUT- Bcl-w 3'-UTR vector using Lipofectamine 2000 (Invitrogen). Forty-eight hours after transfection, cells were collected and analyzed using the Dual-Luciferase Assay System (Promega, Madison, WI, USA). Each assay was performed in triplicate.

### Statistical Analysis

The miR-203 expression in tissue samples was determined as non-normal distribution by using Kolmogorov-Smirnov test. Thus, Mann-Whitney U test or Kruskal-Wallis test was used to evaluate the significance of miR-203 differences between groups. Receiver operating characteristic (ROC) curve was performed to evaluate the discrimination power of miR-203 for discriminating progressed patients from those without progression, and the optimal cutoff value was set based on Youden index (sensitivity+specificity-1)[16]. Survival curves were constructed by Kaplan-Meier method, and compared by log-rank test. Cox model was employed for identification of independent factors of PFS and OS. Student’s t-test was performed to determine significance of data in viability assay, apoptosis assay and luciferase reporter assay. The relationship between miR-203 and target gene expression was explored by Spearman correlation. All statistical analyses were performed using SPSS Statistics 17.0 for Windows (IBM Corporation, Armonk, NY, USA).

## Results

### Patient characteristics

The median age of the 108 BC patients receiving cisplatin-based adjuvant chemotherapy was 62 (range 42–81) years, and the median follow-up period was 51.5 (range 6–65) months. During follow-up, 45 patients showed progression, of whom 32 cases had locoregional recurrence, 11 cases showed distant metastasis, and 39 finally died with BC. The detail baseline characteristics of patients were shown in Table 1.

**Table 1:** Characteristics of 108 bladder cancer patients treated with cisplatin-based adjuvant chemotherapy.

Characteristic	No. (%)
Total	108 (100.0)
Median age (years)	62 (range 42–81)
Median follow-up time (months)	51.5 (range 6–65)
Progression	
No	63(58.3)
Yes	45(41.7)
Mortality	
No	53(49.1)
Yes	55(50.9)
Gender	
Male	83(76.9)
Female	25(23.1)
Grade	
G2	22(20.4)
G3	86(79.6)
T stage	
pT2	18(16.7)
pT3	69(63.9)
pT4	21(19.4)
Nodal status	
Negative	45(41.7)
Positive	63(58.3)
Chemotherapy regimens	
MVAC	73(67.6)
GC	35(32.4)

### Reduced miR-203 expression was associated with disease progression

**Fig 1:**
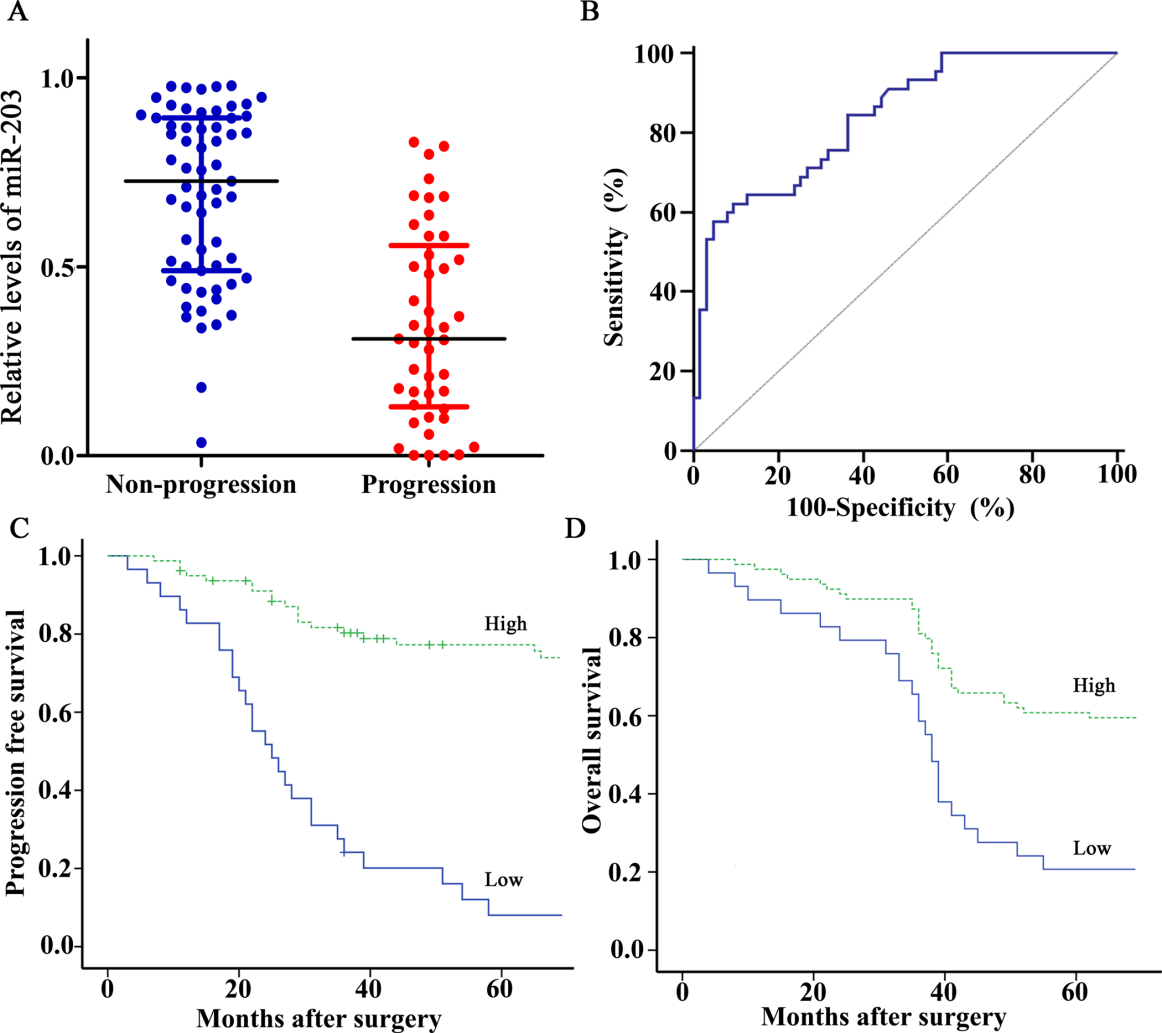
Expression and prognosis of miR-203 for bladder cancer patients treated with cisplatin-based chemotherapy. (A) miR-203 levels were detected by RT-qPCR method and normalized against U6 RNA in 108 cases of bladder cancer tissues. Levels of miR-203 in progression group were significantly lower than those in non-progression group (*P*<0.001, Mann-Whitney U test). (B) ROC curve distinguished patients with progression from those without progression using miR-203, with an area under the ROC curve value of 0.839 (95% CI, 0.756–0.903). (C, D) Kaplan-Meier PFS and OS curves based on miR-203 expression of bladder cancer patients. The optimal cut off value (0.345) calculated by ROC analysis was used to classify the patients as high and low miR-203 expression groups. Low expression of miR-203 was significantly correlated with shortened PFS or OS (Both *P*<0.001, log-rank test).

The miR-203 levels were significantly decreased in progression group compared with non-progression group (*P*<0.001; Fig 1A and S1 File). ROC curves analyses illustrated miR-203 yielded an area under the ROC curve (AUC) value of 0.839 (95% CI, 0.756–0.903) in distinguishing patients with progression from those without progression, which was more accurate than guessing (*P*<0.001; Fig 1B). However, no significant associations were found between miR-203 levels and age, gender, grade, T stage, nodal status, and chemotherapy regimens (all at *P*>0.05; Table 2).

**Table 2:** Associations between miR-203 and clinicopathological characteristics.

Parameters	median (interquartile range)	*P*-value^a^
Age		0.345
<62	0.574(0.353–0.846)	
≥62(median)	0.521(0.242–0.811)	
Gender		0.088
Male	0.581(0.345–0.850)	
Female	0.443(0.248–0.686)	
Grade		0.565
G2	0.628(0.370–0.781)	
G3	0.511(0.331–0.832)	
T stage		0.305
pT2	0.697(0.224–0.819)	
pT3	0.531(0.356–0.859)	
pT4	0.454(0.294–0.673)	
Nodal status		0.068
Negative	0.566(0.209–0.756)	
Positive	0.523(0.382–0.881)	
Chemotherapy regimens		0.803
MVAC	0.515(0.339–0.826)	
GC	0.637(0.281–0.830)	
Progression		<0.001
No	0.727(0.490–0.894)	
Yes	0.309(0.129–0.556)	

^a^ *P*-value was estimated by Mann-Whitney U test or Kruskal-Wallis test.

### miR-203 predicted prognosis in BC patients after chemotherapy

Based on the optimal cut off value (0.345) calculated by ROC analysis for BC progression after chemotherapy, 79 patients whose miR-203 levels were above 0.345 were classified as high miR-203 expression group, the remaining 29 cases were classified as low miR-203 expression group. Kaplan–Meier survival curve showed low expression of miR-203 was significantly correlated with shortened PFS and OS (Both at *P*<0.001; Fig 1C and 1D).

Univariate Cox regression analyses revealed significant associations between miR-203, T stage, nodal status and PFS (all at *P*<0.05; Table 3), as well as miR-203, T stage and OS (all at *P*<0.05; Table 3). When putting above significant factors into multivariate Cox model, miR-203, T stage, and nodal status demonstrated independent prognostic value for PFS (all at *P*<0.05; Table 3), and miR-203, T stage demonstrated independent prognostic value for OS (all at *P*<0.05; Table 3).

**Table 3:** Univariate and multivariate Cox analysis of progression free survival and overall survival for bladder cancer patients.

Parameters	Categories	Univariate analysis		Multivariate analysis	
		HR (95% CI)	*P* value	HR (95% CI)	*P* value
PFS					
Age	<62 *VS* ≥62	1.189(0.661–2.136)	0.563		
Gender	Female *VS* Male	0.703(0.369–1.340)	0.285		
Grade	G2 *VS* G3	1.542(0.688–3.456)	0.293		
T stage	T2 *VS* T3 *VS* T4	1.664(1.006–2.751)	0.047	1.695(1.050–2.737)	0.031
Nodal status	Negative *VS* Positive	2.645(1.340–5.223)	0.005	2.605(1.309–5.184)	0.006
Chemotherapy regimens	MVAC *VS* GC	1.449(0.747–2.808)	0.272		
miR-203 level	Low *VS* High	0.143(0.078–0.264)	<0.001	0.154(0.082–0.288)	<0.001
OS					
Age	<62 *VS* ≥62	1.160(0.683–1.970)	0.583		
Gender	Female *VS* Male	0.861(0.469–1.581)	0.630		
Grade	G2 *VS* G3	1.873(0.847–4.142)	0.121		
T stage	T2 *VS* T3 *VS* T4	1.844(1.184–2.874)	0.007	1.800(1.154–2.807)	0.010
Nodal status	Negative *VS* Positive	1.385(0.804–2.388)	0.241		
Chemotherapy regimens	MVAC *VS* GC	1.516(0.826–2.782)	0.180		
miR-203 level	Low *VS* High	0.349(0.203–0.600)	<0.001	0.359(0.209–0.616)	<0.001

Abbreviations: PFS, progression free survival; OS, overall survival; HR, Hazard ratio; CI, Confidence interval

### Overexpression of miR-203 enhanced cisplatin sensitization on BC cells

**Fig 2:**
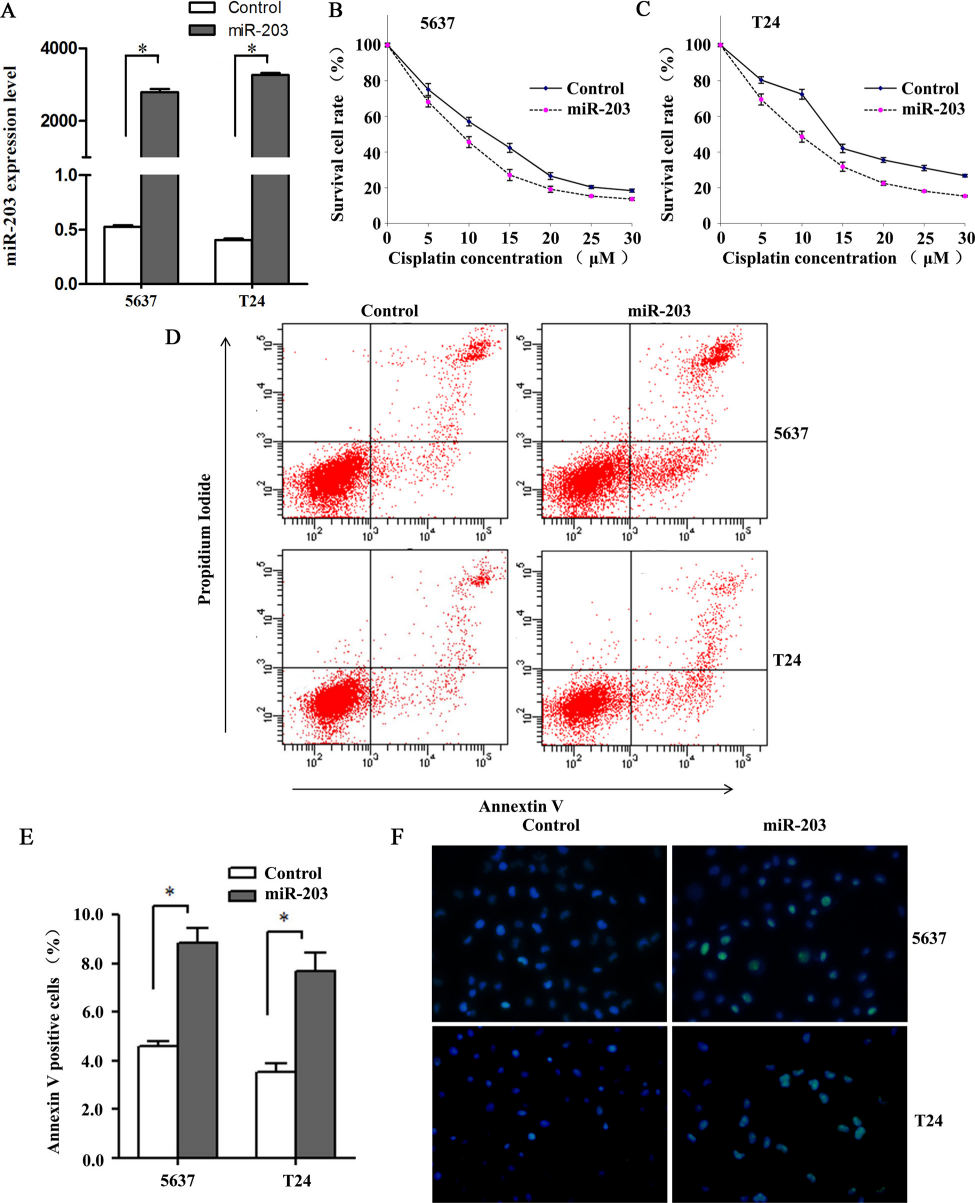
miR-203 enhances cytotoxicity of cisplatin on 5637 and T24 bladder cancer cells. (A) miR-203 expression levels in 5637 and T24 cells transfected with miR-203 mimics/negative control. miR-203 levels were significantly increased in cells transfected with miR-203 mimics compared with cells transfected with negative control (*P<0.001, t test, n = 6). (B, C) Concentration-dependent curves for 5637 and T24 cell lines transfected with miR-203 mimics and negative control at 24h. Cell viabilities of miR-203-overexpressing cells were dramatically reduced when compared with negative control cells at 5, 10, 15, 20, 25 and 30μM cisplatin (all at *P*<0.05, t test). (D) Flow cytometry analysis for apoptosis via double staining of cells with Annexin V FITC and propidium iodide (PI). (E) Quantification analysis of apoptosis shown in Fig 2B. Overexpression of miR-203 significantly augmented apoptosis in 5637 and T24 cell lines (*P<0.01, t test, n = 6). (F) TUNEL assay indicated 5637 and T24 cell lines transfected with miR-203 mimics showed elevated levels of DNA cleavage compared with normal control (40×). Cells were stained with DAPI and subjected to TUNEL assay to detect DNA and apoptotic cells.

To explore the effect of miR-203 on cisplatin chemosensitivity in BC cells, concentration-dependent curves for 5637 and T24 BC cell lines transfected with miR-203 mimics and negative control were plotted. As shown in Fig 2A, 24 hours after transfection, the miR-203 expression levels in 5637 and T24 cells transfected with miR-203 mimics were 5297 and 8089 times higher than those transfected with negative control (*P*<0.001). Cell viabilities of miR-203-overexpressing 5637 and T24 cells were dramatically reduced when compared with cells transfected with negative control at a concentration gradient of 5, 10, 15, 20, 25 and 30μM for 24h (all at *P*<0.05; Fig 2B and 2C). The half maximal inhibitory concentration (IC50) values of cisplatin for 5637 and T24 cells were 11.1(95%IC 9.7–12.5) μM and 14.3(95%IC 12.7–16.1) μM, respectively. Overexpression of miR-203 significantly decreased the IC50 values to 8.3(95%IC 6.9–9.6) μM and 9.0(95%IC 7.6–10.4) μM, respectively. In addition, the effects of miR-203 on gemcitabine and cisplatin combination were shown in S2 File.

Then, we performed a flow cytometry analysis to quantify apoptosis via double staining of cells with annexin V FITC and propidium iodide (PI). The results demonstrated overexpression of miR-203 significantly augmented apoptosis in both BC cell lines (both at *P*<0.05; Fig 2D and 2E). To verify that the increase in apoptosis was due to the activation of the above-mentioned apoptosis pathway, we measured DNA fragmentation by TUNEL assay and confirmed that cells transfected with miR-203 showed elevated levels of DNA cleavage (Fig 2F).

### Bcl-w and Survivin were direct downstream targets of miR-203

**Fig 3:**
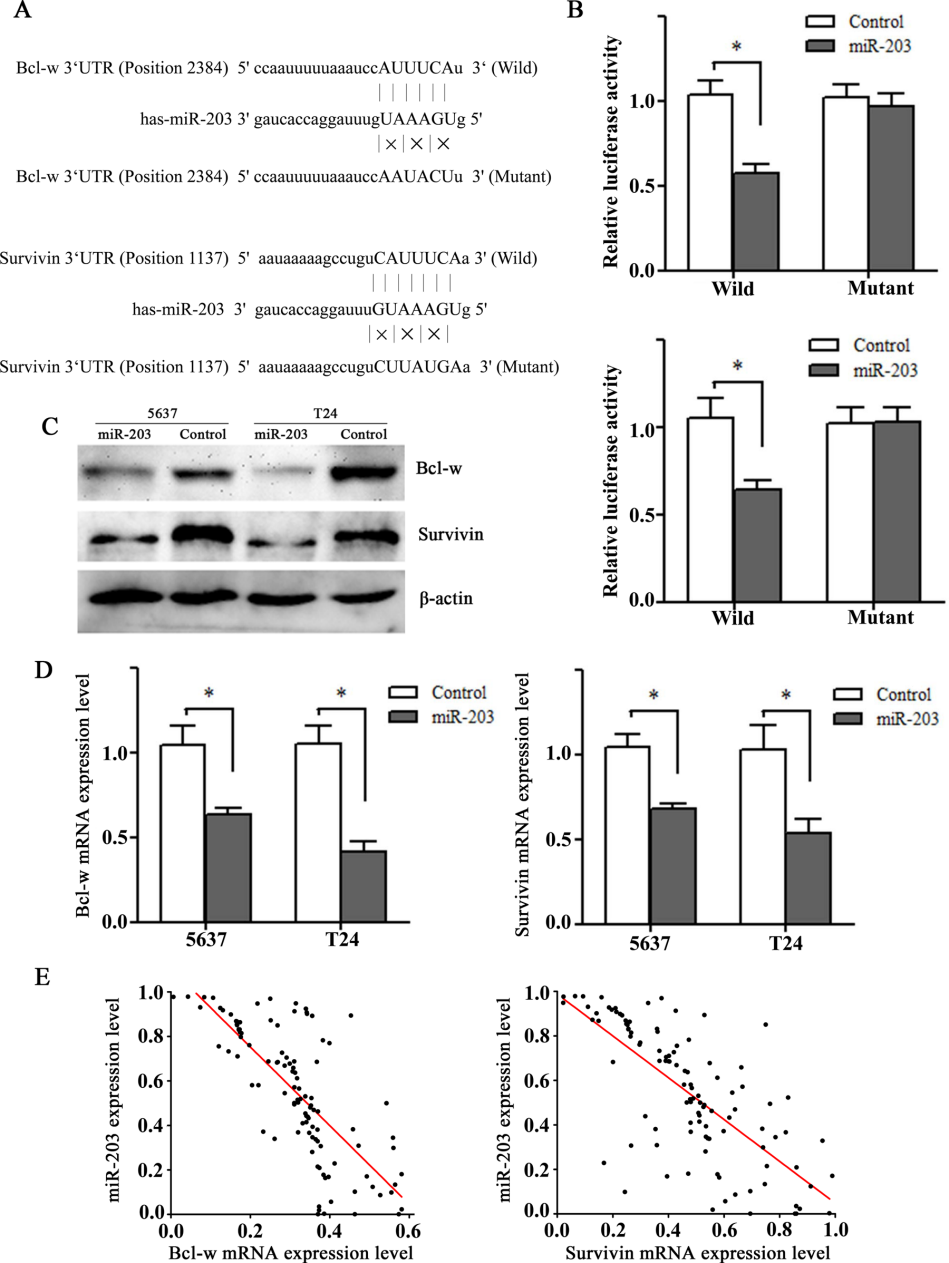
Bcl-w and Survivin are direct downstream targets of miR-203. (A) Illustration of the predicted miR-203 targeting sites in 3’UTR regions of Bcl-w and Survivin. (B) Dual-Luciferase activity assay was performed in HEK293T cells co-transfected with control/ miR-203 and pmiR-REPORT vectors with wild-type/mutant 3′-UTR of Bcl-w (above) and Survivin (below). *P<0.01, t test, n = 6. (C) Western blots showing downregulation of Bcl-w and Survivin proteins after transfection of miR-203 mimics in 5637 and T24 cell lines. β-actin was used as a control. (D) RT-qPCR showing downregulation of Bcl-w and Survivin mRNAs after transfection of miR-203 mimics in 5637 and T24 cell lines. *P<0.01, t test, n = 6. (E) Spearman’s correlation analysis showing a significant inverse association between miR-203 and Bcl-w mRNA or Survivin mRNA expression in bladder cancer tissues(r = -0.781, -0.740, both at *P*<0.001).

Bcl-w and Survivin were predicted as potential direct targets of miR-203 by two major target prediction programs, TargetScan 5.1 (http://www.targetscan.org/) and miRanda (http://www.microrna.org/). Moreover, Bcl-w and Survivin played important roles in BC development, and their downregulation were associated with enhanced sensitivity to cisplatin-induced apoptosis[17–20]. The predicted miR-203 targeting sites in 3’UTR regions of Bcl-w (also known as BCL2L2) and Survivin (also known as BIRC5) were shown in Fig 3A. A dual-reporter luciferase assay was employed to confirm whether the 3’UTR of Bcl-w and Survivin mRNAs had target sites for miR-203. We constructed a luciferase reporter vector encoding the wild-type 3′-UTR sequence of Bcl-w mRNA or Survivin mRNA, including the predicted miR-203 target sites, and a corresponding mutant reporter vector. As shown in Fig 3B, miR-203 significantly inhibited luminescence intensity of WT-Bcl-w 3'-UTR vector and WT-Survivin 3'-UTR vector (both *P*<0.01), while mutation of the binding site blocked the decrease of luciferase activity, indicating there was a direct interaction between miR-203 and the 3’UTR of Bcl-w mRNA or Survivin mRNA.

To further confirm their relationship in BC, we transfected 5637 and T24 cell lines with miR-203 mimics or negative control, and analyzed the mRNA and protein expression of Bcl-w or Survivin by RT-qPCR and western-blotting assays. The results showed that endogenous expressions of Bcl-w and Survivin were significantly decreased in both mRNA and protein levels for miR-203 transfected BC cells (Fig 3C and 3D). Consistent with this, there was also a significant inverse association between miR-203 and Bcl-w or Survivin mRNA expression in BC tissues (r = -0.781, -0.740, both at *P*<0.001; Fig 3E). Taken together, miR-203 might exert its function by directly targeting Bcl-w and Survivin gene in BC.

## Discussion

Postoperative cisplatin-based chemotherapy has been widely used for muscle invasive or metastatic BC, resulting a response in up to 70% patients[21]. Unfortunately, due to chemoresistance of cancer cells, the response is not sustained in more than 50% of cases, resulting in a 5-year survival rate of 15%[22, 23]. Thus, it is important to better understand the factors that determine chemotherapy response and prognosis. Nordentoft et al[24] have found 15 miRNAs in response to cisplatin treatment, and 5 miRNAs associated with survival time, of which 3 miRNAs (miR-886-3p, miR-923, miR-944) were considered as predictors of both cisplatin response and prognosis. Previous studies have documented decreased expression of miR-203 was associated with tumor progression and poor prognosis in some cancers, such as human head and neck squamous cell carcinoma[25], gliomas[26], and esophageal adenocarcinoma[27]. Consistent with the findings described above, this study quantified miR-203 levels in a cohort of BC patients treated with radical cystectomy and cisplatin-based adjuvant chemotherapy, and found miR-203 was significantly reduced in patients with progressive disease, while not associated with other clinicopathological characteristics. Moreover, ROC analysis showed miR-203 had some diagnostic power in discriminating patients with or without progression. Based on the optimal cutoff value of miR-203, we classified all patients into high and low miR-203 expression groups. Kaplan–Meier analysis demonstrated low miR-203 expression group had poor PFS and OS. Multivariate Cox regression analyses demonstrated that miR-203 was an independent prognostic factor for BC patients. Thus, mi-203 could be used for prognosis prediction of BC patients treated with cisplatin-based adjuvant chemotherapy.

Cellular resistance to cisplatin often exhibits a multifactorial nature, including weakened intracellular drug uptake, increased DNA damage repair, and decreased execution of the apoptotic program [28]. Drayton et al[29] found miR-27a contributed to cisplatin resistance through regulation of intracellular glutathione. Vinall et al[30] showed co-treatment with miR-34a and cisplatin resulted in a dramatic inhibition in proliferation of cancer cells compared to treatment with cisplatin alone. Conversely, cisplatin induces demethylation of the miR-34a promoter and increases miR-34a expression, further sensitizing BC cells to cisplatin[31]. In this study, we found that transfection of BC cells with miR-203 mimics sensitized cells to the cytotoxic activity of cisplatin. Meanwhile, data from apoptosis assays showed when adding 5μM cisplatin, more apoptosis was found in miR-203-overexpressing bladder cancer cells, suggesting that the inhibition of BC cells viability might be related at least in part, to the enhanced susceptibility to cisplatin-induced apoptosis. Thus, the combination regimen of miR-203 mimics and cisplatin might improve BC patient treatment outcomes.

To gain a further understanding of the role of miR-203 in BC development, its potential downstream targets were analyzed. As a member of the Bcl-2 family, Bcl-w can promote cell survival by inhibiting the intrinsic pathway of apoptosis[32]. Chen et al[33] found Bcl-w was over-expressed in BC specimens compared to adjacent normal tissues, suggesting it played an important role in carcinogenesis of BC. Survivin, which was a key member of the inhibitor of apoptosis protein (IAP) family[34], executed its antiapoptotic function by blocking caspases activity in a complex with X-linked inhibitor of apoptosis protein (XIAP)[35]. A multicenter study found Survivin expression was associated with an elevated risk of BC recurrence and cancer-specific mortality[36]. Our data demonstrated that miR-203 could directly bind the 3’-UTR of both Bcl-w and Survivin, resulting in down-regulated expression of Bcl-w and Survivin at post-transcriptional level. These were supported by clinical data where we found miR-203 expression was and negatively correlated with Bcl-w and Survivin mRNAs expression in BC tissues samples. Thus, miR-203 could be considered as a potential therapeutic agent by simultaneous inhibition of antiapoptotic factors Bcl-w and Survivin.

In conclusion, our study demonstrates that decreased miR-203 can be used as a predictor for progression and prognosis of BC patients treated with cisplatin based chemotherapy. Moreover, overexpression of miR-203 can enhance cisplatin sensitization by promoting apoptosis via directly targeting Bcl-w and Survivin. Additional multicenter studies are needed to confirm whether miR-203 could be useful as an indicator of chemosensitivity to cisplatin-based chemotherapy regimens as well as a therapeutic target for BC in the clinic.

## Supporting Information

S1 File The miR-203 expression levels of NMIBC patients, Progression group with 45 cases and non-progression group with 67 cases. (XLSX)

S2 File The effects of miR-203 on gemcitabine and cisplatin combination. (DOCX)
