# Supplementary material for: MicroRNA-203 Is a Prognostic Indicator in Bladder Cancer and Enhances Chemosensitivity to Cisplatin via Apoptosis by Targeting Bcl-w and Survivin
Source: PLoS One. 2015 Nov 23;10(11):e0143441. doi: 10.1371/journal.pone.0143441 (PMC4657877; doi:10.1371/journal.pone.0143441)
Supplement: S2 File — (DOCX) [file pone.0143441.s002.docx]

**S2 File. The effects of miR-203 on gemcitabine and cisplatin combination.**

5μM cisplatin and 100nM gemcitabine were used to observe whether miR-203 sensitized the cells to gemcitabine and cisplatin. 5μM cisplatin was selected because it was the minimum concentration that caused statistical difference in cell viability between miR-203-overexpressing cells and negative controlled cells in this study (see Figure 2B and 2C; also presented as following Supplementary Figure 1A). And, 100nM gemcitabine was chosen according to Pinto-Leite et al[1], which was IC35 dose in 5637 and T24 cells.

**Method**

5637 and T24 bladder cancer cells (5×10^3^ cells/ well) were seeded in 96-well plates in triplicate after transfection 24h, and incubated with 100nm gemcitabine+ 5μM cisplatin or 100nM gemcitabine only in 100 μl culture medium for another 24h.

**Results**

The results showed cell viabilities of miR-203-overexpressing 5637 and T24 cells were dramatically reduced when compared with negative controlled cells at 100nm gemcitabine + 5μM cisplatin (Supplementary Figure 1C), while no signiﬁcant effect was observed at 100nm gemcitabine only (Supplementary Figure 1B). Moreover, simultaneous treatment with cisplatin and gemcitabine resulted less survival rate than only treatment with 5μM cisplatin for miR-203-overexpressing 5637 and T24 cells. Taken together, we found miR-203 resulted in the lowest survival rate to cells treated with cisplatin and gemcitabine, and the reduced survival rate was then observed for cells treated with cisplatin alone, and no effect on cells treated with gemcitabine alone.

**Discussion and Conclusion**

Data from results showed miR-203 might not be a direct sensitizer for gemcitabine. In addition, Zhou et al [2] have found exogenous expression of miR-203 in chemo-naïve colorectal cancer cells induced oxaliplatin resistance. Therefore, we concluded that miR-203 might not function as a general sensitizer for all chemotherapy, and detailed mechanism of its sensitizing effect for some special chemotherapy drugs is worth further study.


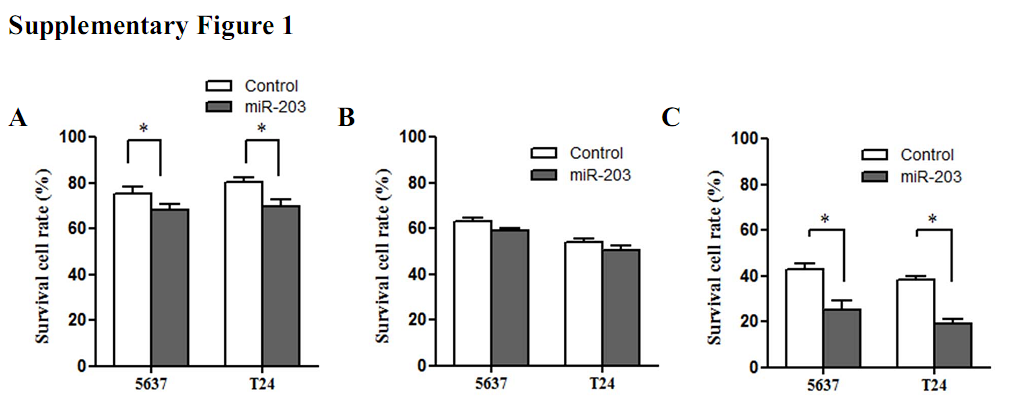


Supplementary Fig.1 Cell viabilities of 5637 and T24 cell lines transfected with negative control and miR-203 mimics in cisplatin (A), gemcitabine (B), and cisplatin and gemcitabine combination (C).

**References**

[1] Pinto-Leite R, Arantes-Rodrigues R, Ferreira R, Palmeira C, Colaco A, Moreira da Silva V, et al. Temsirolimus improves cytotoxic efficacy of cisplatin and gemcitabine against urinary bladder cancer cell lines. Urologic oncology. 2014;32(1):41 e11-22.

[2] Zhou Y, Wan G, Spizzo R, Ivan C, Mathur R, Hu X, et al. miR-203 induces oxaliplatin resistance in colorectal cancer cells by negatively regulating ATM kinase. Molecular oncology. 2014;8(1):83-92.
